# Adaptation of an international virtual patient collection to the COVID-19 pandemic

**DOI:** 10.3205/zma001385

**Published:** 2020-11-03

**Authors:** Inga Hege, Malgorzata Sudacka, Andrzej A. Kononowicz, Julia Nonnenmann, Julia Banholzer, Jörg Schelling, Martin Adler, Bernarda Espinoza, Marie Astrid Garrido, Katja Radon

**Affiliations:** 1 Universität Augsburg, Med. Fakultät, Medical Education Sciences, Augsburg, Germany; 2 Klinikum der LMU München, Institut für Didaktik und Ausbildungsforschung in der Medizin, München, Germany; 3 Jagiellonian University Medical College, Faculty of Medicine, Institute of Medical Education, Krakow, Poland; 4 Jagiellonian University Medical College, Faculty of Medicine, Department of Bioinformatics and Telemedicine, Krakow, Poland; 5 LMU Munich, Faculty of Medicine, Munich, Germany; 6 Instruct gGmbH, Munich, Germany; 7 Klinikum der LMU München, Center for International Health, Munich,Germany; 8 Klinikum der LMU München, Institut und Poliklinik für Arbeits-, Sozial- und Umweltmedizin, Munich,Germany

**Keywords:** virtual patients, COVID-19, clinical reasoning, internationalization

## Abstract

The COVID-19 pandemic posed new global challenges for teaching. We met these challenges as an international collaboration by adapting a collection of virtual patients for clinical reasoning training to this novel context.

## Zusammenfassung

Die COVID-19-Pandemie stellt den Unterricht vor neue globale Herausforderungen. Wir haben uns diesen Herausforderungen in internationaler Zusammenarbeit gestellt, indem wir eine Sammlung virtueller Patient*innen für das Üben von klinischem Entscheiden (clinical reasoning) an diesen neuen Kontext angepasst haben.

## Background

Virtual patients (VPs) are online cases often used to train health profession students in clinical reasoning. This includes gathering information, formulating and prioritizing differential diagnoses, ordering tests to confirm or rule out diagnoses, deciding about a final diagnosis and developing a treatment plan [1]. Despite technological advancements, VPs are still quite static and cannot yet be rapidly or even automatically adapted to changing contexts. Thus, COVID-19 posed several new challenges to authors and curators of VP collections. First, naturally VPs were designed to take place in a non-pandemic environment, and do not reflect this new contextual factor. So, in the current situation the clinical reasoning process has changed in a way that first of all it has to be pre-assessed whether a patient might be infected by COVID-19 and protective measures have to be undertaken. 

Second, for all VPs showing COVID-19- like symptoms this is a new differential diagnosis that has to be considered. Risk factors, like exposure or comorbidities, influence the whole process of clinical reasoning and the probability of a COVID-19 diagnosis has to be considered based on the region in which the VP scenario takes place.

Third, due to the global need for digital teaching in healthcare education the demand for using VP collections has risen dramatically in spring 2020. This need included a demand of VPs in new languages, such as Spanish and Portuguese including adaptations to local environments, such as Latin American countries strongly affected by the crisis where the demand on COVID-19 knowledge is especially visible in recorded e-learning activities [2]. 

## Project description

For our publicly available collection of 75 VPs in German, English, and Polish [https://crt.casus.net/] which was available in the VP system CASUS at the beginning of the pandemic, it was neither possible nor reasonable to adapt all VPs to the new context in this rapidly changing situation and the short timeframe available to implement changes. Additionally, data on COVID-19 was often inaccurate and changing regularly in the first weeks of the pandemic.

Therefore, our approach was based on the following four actions:

1. Updating selected VPs with similar key-symptoms, such as a virtual patient with a common cold suffering from fever and cough, to the current situation without changing the final diagnoses to represent a realistic probability of diseases.
2. Creating and translating two new VPs who actually are tested positive for COVID-19.
3. Providing an introduction to the VP collection explaining that the VPs have been designed for a non-pandemic context. Additionally, we provide further national and international information sources about COVID-19 for the learners.
4. Expanding the VP collection by translating a subset into Spanish and Portuguese and adapting them if necessary, to local conditions.

## Results

### Process

A major challenge for adapting and designing the VPs for an international group of learners were the constantly changing guidelines on COVID-19, which differ between countries, regions and even hospitals. Also, often decisions depend on the circumstances such as availability of testing facilities and equipment or hospital regulations. Since it is not possible to factor in all these variations, we solved this by asking open questions concerning the process and providing feedback by linking to up-to-date guidelines from global institutions such as the World Health Organization (WHO) [https://www.who.int/emergencies/diseases/novel-coronavirus-2019] or the European Centre for Disease Prevention and Control (ECDC) [https://www.ecdc.europa.eu/en/covid-19-pandemic]. Additionally, we encourage learners to look for more specific information on the current process in their area.

Another challenge was the provision of updated multimedia material for the VPs to illustrate the current situation, for example by showing healthcare professionals with protective gear. With the restricted access to hospitals and doctor's offices it was challenging to get such authentic multimedia material. We have not yet a final solution for that, but experiment with publicly available images and editing the images used so far. 

#### Increase in use

**Figure 1:**
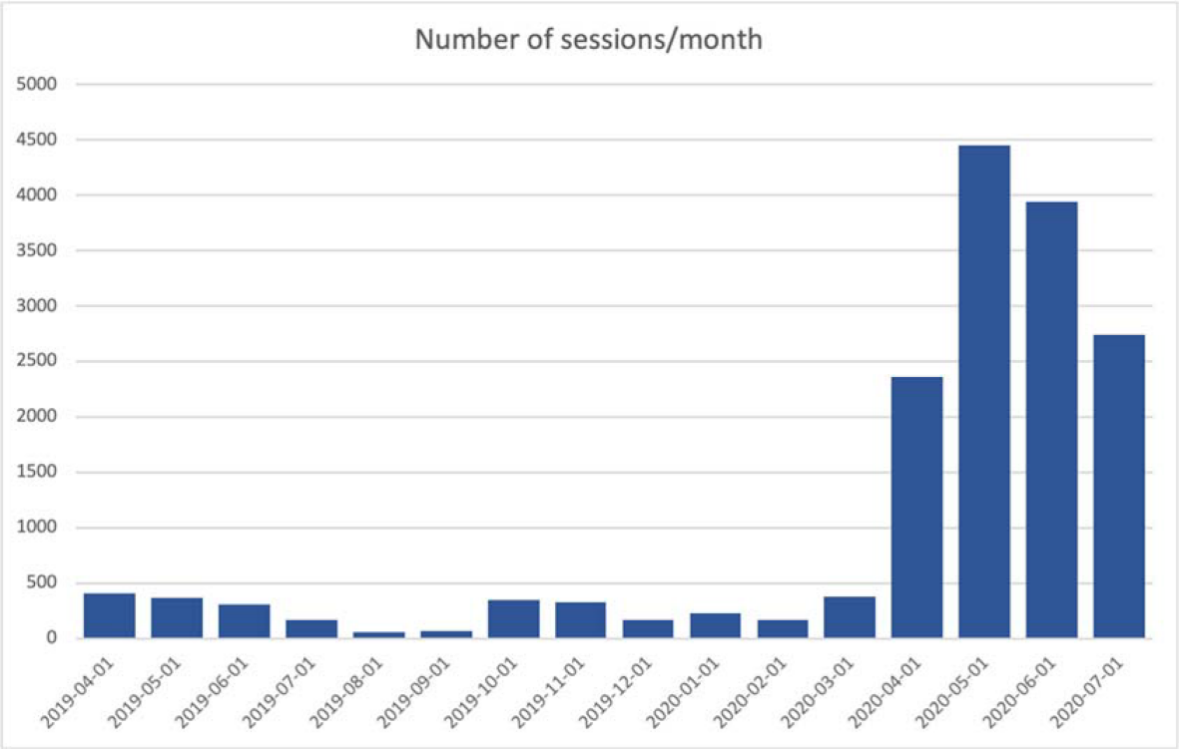
Increase in number of sessions/month for the publicly open clinical reasoning VP courses in German, English, Polish, and Spanish since April 2019.

Since April 2020 we recognized a major increase in inquiries to use the VP collections in medical curricula (see figure 1 (Fig. 1)). This included more learners from medical schools within Europe, but also new users from Latin America. 

## Discussion and conclusions

This rapid global change of the context in which teaching and clinical reasoning takes place requires manifold and quick adaptations of the teaching environment. On the other hand, it provides a great opportunity for students to experience how context influences the clinical reasoning process [3], which we aimed to demonstrate with the adaptations to the VP collection. 

In the future, a more dynamic adaptation of virtual patients to contextual factors is desirable. This might not only help in such extreme situations but also provide in general a more diverse and adaptable learning experience for learners. 

The substantial increase in using the VP collection was potentially caused by the fact that most medical schools prohibited face-to-face teaching and especially patient contacts. Solving virtual patients gives an opportunity for medical schools to document obtaining required learning objectives related to clinical presentations which were impossible to reach otherwise due to unexpected hindrances [4]. Hopefully, the VP collections will be used and integrated into curricula even when face-to-face teaching with patients is possible again, not as a replacement for bedside teaching, but as a valuable addition for training in a safe environment.

Therefore, we sustain the international collaboration to further expand the VP collection to provide a source for deliberate practice of clinical reasoning. 

## Funding

The original creation of the VP collections in German and English was funded by a Marie Sklodowska-Curie Global Fellowship (No 654857). The adaptations were supported by the Erasmus+ Knowledge Alliance DID-ACT (612454-EPP-1-2019-1-DE-EPPKA2-KA). The translations and adaptations to the Latin American context were supported by the German Academic Exchange Service within the exceed program funded by the German Federal Ministry for Economic Cooperation and Development. The Polish translations at the Jagiellonian University were financed from internal funds (N41/DBS/000207).

## Acknowledgements

We thank all educators and clinicians who were involved in the creation, translation, review, and adaptation of the virtual patients, especially, Leonardo Briceno (Universidad del Rosario, Bogotá, Colombia), María Teresa Solis Soto (USFX, Sucre, Bolivia) and Claudia Meneses (USAC, Guatemala City, Guatemala) who coordinated these efforts locally. Without them this quick transformation would not have been possible. 

## Competing interests

Martin Adler is CEO of the Instruct gGmbH which develops the virtual patient system CASUS. 

The authors declare that they have no competing interests.
